# Phylodynamic Analysis and Implication of HCV Genotype 4 Variability on Antiviral Drug Response and T-Cell Recognition

**DOI:** 10.3390/v12121363

**Published:** 2020-11-28

**Authors:** Giuseppina Maria Elena Colomba, Noemi Urone, Vito di Marco, Donatella Ferraro

**Affiliations:** Dipartimento di Scienze per la Promozione della Salute, Materno-Infantile di Medicina Interna e Specialistica di Eccellenza “G. D’Alessandro”, 90133 Palermo, Italy; giuseppina.colomba@gmail.com (G.M.E.C.); noemi.urone@libero.it (N.U.); vito.dimarco@unipa.it (V.d.M.)

**Keywords:** phylodynamic, HCV, genotype 4, DAA, Bayesian analysis, viral epitopes, genetic variability, RAS, tMRCA, T cell recognition

## Abstract

Therapies for HCV care could change the prevalence and the geographic distribution of genotypes due to differences in Sustained Virologic Response (SVR). In this scenario, uncommon genotypes/subtypes, such as genotype 4, could spread from high-risk groups, replacing genotypes eradicated by antiviral drugs. Genotype eradication is also strongly influenced by the CD8+ T cell response. In this study, the genetic variability in HCV genotype 4 strains obtained from a cohort of 67 patients naïve to DAA therapy was evaluated. We found that the presence of resistance-associated substitutions (RAS) was able to affect drug responses. Next, using a prediction tool, viral mutations were identified by their ability, or lack thereof, to reduce the binding affinity with HLA, which affects T cell recognition. The Bayesian coalescent analysis suggested two different circulation clusters, one in risk groups (IDUs and MSM) and the other due to migration flows, dated to 1940 and 1915, respectively. Most of the RAS overlapped with HLA and a lack of binding mutations was observed in 96% of strains. This study describes the introduction of HCV genotype 4 in a region of the Mediterranean basin and evaluates how HCV genotype 4’s genetic variability could affect the response of antiviral drugs and CD8+ T cell recognition.

## 1. Introduction

The hepatitis C virus (HCV) is the leading cause of liver disease with over 170 million people infected. HCV is classified by eight genotypes (1–8) and 90 subtypes according to the International Committee on Taxonomy of Viruses (ICTV). HCV genotype 4 (HCV-4) accounts for 12–15% of worldwide HCV infections (15–18 million), showing a prevalence of 1% and 10–20% in the USA and Europe, respectively. It dominates in North-Central Africa and in Arabic countries, such as Egypt, Lebanon, Syria, Saudi Arabia, and Kuwait [1,2], where a large population is HIV/HCV co-infected and access to care is limited [3]. HCV-4 is remarkably heterogeneous, and it is classified into 18 confirmed subtypes whose prevalence varies geographically. It is thought that HCV-4 originated in Central and Western Africa and then spread to neighboring regions. In Egypt, subtype 4a accounts for the majority of HCV infections and its high prevalence is probably due to the unsafe use of injections during the past anti-schistosomal public health campaigns [4]. Southern Europe countries, particularly France, have reported the spread of subtypes 4a and 4d due to immigration and the movement of intravenous drug users (IDUs) across European borders [5,6,7]. HCV-4 prevalence is increasing in Italy and subtype 4d seems endemic in southern regions. Phylogenetic analysis conducted in the Calabria region on HCV-4d, with a prevalence of 8.7%, dated the introduction of the virus to sometime between the two World Wars, probably imported from Africa during the Italian colonial period (1922–1940) [8].

In Sicily, HCV-1b predominates with 70% of infections [9]. As supported by phylogenetic analysis conducted among the people of a small Sicilian town, HCV-1b was introduced in the 1940s and disseminated through iatrogenic transmission, such as the use of glass syringes, transfusions, and unsafe health care [10]. According to the latest European Association for the Study of the Liver guidelines, among patients infected with HCV genotype 4, the SVR rate after genotype-specific therapy is 96% for the Sofosbuvir/Ledipasvir or Grazoprevir/Elbasvir regimen [11]. In this scenario, novel therapeutic regimes, based on direct antiviral agents (DAAs), could change both the prevalence of HCV infection and the geographic distribution of the different genotypes. Uncommon HCV genotypes/subtypes such as HCV-4 could spread from high-risk groups replacing the endemic genotypes eradicated by antiviral drugs. Additionally, virus eradication after antiviral therapy is strongly influenced by the CD8+ T cell responses targeting multiple viral epitopes bound to human leukocyte antigen class I (HLA-I) [12]. This is the first study that describes the phylogenetic reconstruction of HCV genotype 4 in Sicily, highlighting the circulation of different lineages through Bayesian coalescent analysis and describing how the genetic variability of HCV-4d isolated from patients naïve to DAA therapy can affect antiviral drugs’ responses and CD8+ T cells’ recognition.

## 2. Materials and Methods

From 1997 to 2018, HCV genotypes were identified with reverse hybridization in a cohort of 5197 patients attending the General Hospital of Palermo. Sixty-seven Sicilian patients, 33 women and 34 men, chronically infected by HCV-4 and naïve to DAA therapy were included in this study. Fourteen patients were IDUs and 11 of them were HIV co-infected.

To estimate the genetic variability and evaluate the presence of resistance-associated substitutions (RAS), new primers for the NS3 region (step I: 4NS3_OF GGCACCATAATCACAAGCCTCACCG and 4NS3_OR GGCRACTTGGTAYGTCTGGGGCAC, step II: 4NS3_IF CGGCAGAGAYACCAAYGAGAACTG and 4NS3_IR GCDGGAGGAGTRGARTTGTCAGAGAA) and the NS5A region (step I: 4NS5A_OF TCATCCCTSACTGTGACNTC and 4NS5A_OR TCYACCTCNGTGAAGAAYT, step II: 4NS5A_IF CTCAGACGNCTCCACAAGT and 4NS5A_IR GCAGGGGCAYTTGATGTT) were designed. All known RASs were inside the amplified NS3 and NS5A regions, and the more-variable NS5B region (450bp) was amplified only for Bayesian analysis, as previously described [13]. NS3 and NS5A sequences were published in GenBank under accession numbers KY460555 to KY460621 and KY460622 to KY460673, respectively.

The time of the most recent common ancestor (tMRCA) was investigated with demographic models of population growth with constant size, exponential growth, and a Bayesian skyline (BSP). These models were compared to a marginal likelihood estimation (MLE) with path sampling and stepping stone analysis implemented in Bayesian Evolutionary Analysis by Sampling Trees (BEAST 2) software. The exponential growth model fit best. To increase the number of parsimony-informative sites, the NS3 and NS5B sequences were concatenated (total 875 bps). The sequences were tagged with the sampling dates (1998–2015) and were run in BEAST 2 for at least 50 million generations and sampled every 5000 states by a Markov chain Monte Carlo (MCMC) analysis. All BEAST output log files were analyzed with the TRACER v1.6.0 program until the effective sample size (ESS) numbers were >250. A maximum clade credibility (MCC) tree was obtained by summarizing trees using Tree Annotator. The phylogenetic trees were displayed using the FigTree program [14].

Shannon entropy (ES) was used to characterize amino acid variability [15]. To obtain statistical confidence, we used the Monte Carlo randomization strategy. The maximum ES value depends on the number of discrete variables in a sample set. The mixed effects model of evolution (MEME), a maximum likelihood approach that counts the ratio of non-synonymous/synonymous changes (dN/dS), was used to investigate amino acid positive selection [16]. The presence of RASs was evaluated with the Geno2pheno-algorithm [17]. According to the literature, RAS amino acid residues 36, 43, 54, 55, 56, 80,122, 138, 155, 156, 158, 168, and 170 of the NS3/4 protein and 28, 30, 31, 54, 58, 62 and 93 of the NS5A protein were evaluated [18,19]. RAS at position 107 in NS3 proteins and 34, 37, and 47 in NS5A proteins were previously identified in HCV-4-infected patients at failure therapy and were included in the analysis [20,21]. The genetic barrier for RAS selection was analyzed through a minimal score calculation (m.s.), which gives a score of 1 to each nucleotide transition and 2.5 to each nucleotide transversion [22,23].

Known HLA-I restricted HCV epitopes were downloaded from the Immune Epitope Database and Analysis Resource (IEBD) [24] and mapped over previously obtained NS3 and NS5A sequences. IEBD prediction tools were used to identify MHC-I molecules able to recognize HCV epitopes previously described in humans. Next, the allele frequencies were evaluated [25]. The HLA-I binding affinity of wild-type and mutated epitopes was investigated, setting a cutoff of inhibitory concentration equal to 50 nM (IC50) and using an artificial neural network (ANN) score as a binding prediction method. Values <50 nM were considered high affinity, values <500 nM were considered intermediate affinity, values <5000 nM were considered low affinity, and values >5000 nM were considered to have caused a lack of binding [26].

## 3. Results

### 3.1. Phylogenetic Analysis of HCV Isolates

**Figure 1:**
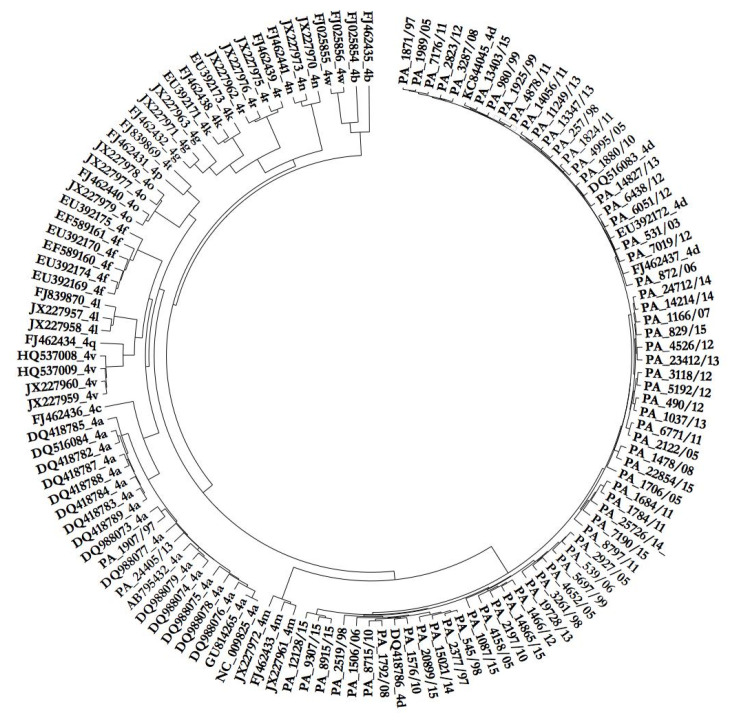
Phylogenetic tree of the NS3 region (450 bp) made with T93+G+I (bootstrap 1000 replicates) containing 67 sequences from Direct Acting Antivirals (DAA) naïve patients (PA number/year) and 64 sequences from HCV-4 with subtypes from *a* to *w* downloaded from GenBank.

Over the last ten years, HCV genotype 4 was identified with reverse hybridization in 172 serum samples (3.3%) from a cohort of 5197 HCV-infected patients who attended the General Hospital of Palermo. From 1997 to 2006 and from 2007 to 2018, genotype 4 prevalence has changed, especially in young men: from 1.8 to 8.6% and from 1.3 to 7.7% in men aged 26–35 and 36–45 years, respectively. HCV genotype 4 counted for 15% of HIV co-infection observed from 2010 to 2018. Sequences of the NS3 gene were obtained from 67 patients chronically infected with HCV genotype 4. Phylogenetic analyses were generated with Tamura’s 3-parameter (T93) model using a gamma (G) distribution and assuming that a certain fraction of sites were evolutionary invariable (I). Using all 18 HCV-4 subtypes as a reference, we demonstrated that all sequences studied belonged to subtype 4d, except two isolates classified as HCV-4a (Figure 1).

### 3.2. Phylodynamic Analysis

**Figure 2:**
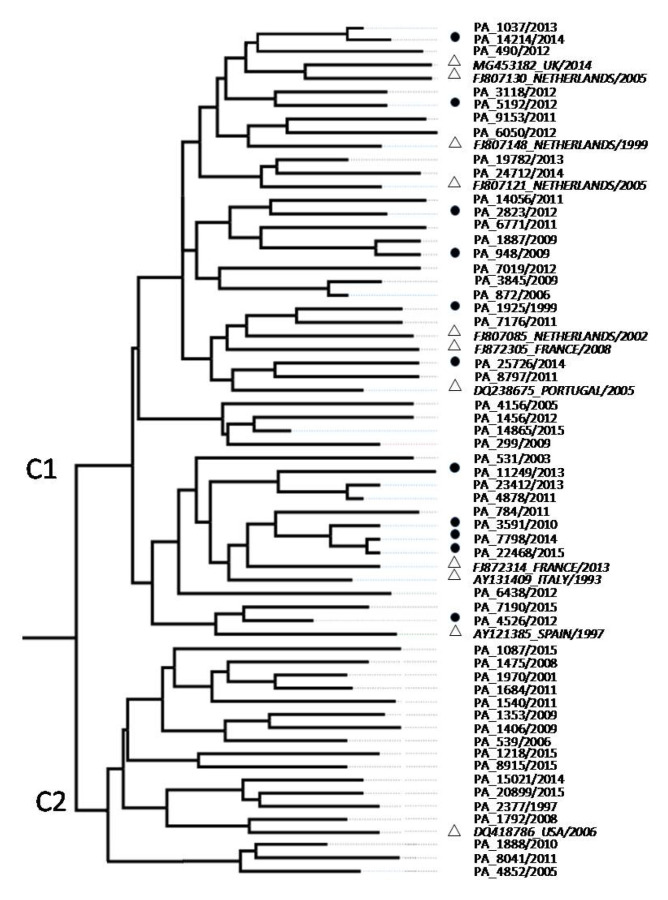
NS5B region (425 bp) maximum clade credibility tree (Tamura-Nei ’93 model, visualized with FigTree software). White triangles represent HCV-4d sequences from GenBank (accession number_country/year) and the black circles represent Sicilian sequences (PA_number/year) obtained from HIV co-infected patients.

Among the 67 infected patients, we only obtained 53 NS5B sequences due to a low viral load or unavailability of the serum. Because previous studies have reported the spread of subtypes 4a and 4d in the risk group, we built a phylogenetic tree using 11 sequences isolated from IDUs, MSM, and North-African immigrants. The MCC showed two clusters (C1 and C2) with a posterior probability limit of 0.9 (Figure 2).

The C1 cluster contained 36 isolates obtained from 31 (86.1%) men (mean 45 years old ± SD 15.7) and 30.5% of them were IDUs and HIV-coinfected (10 men and 1 woman). The C2 cluster included 17 isolates from mono-infected subjects (mean 58 years old ± SD 6.3): eight (47.1%) men and nine (52.9%) women (as shown in Table 1).

**Figure 3:**
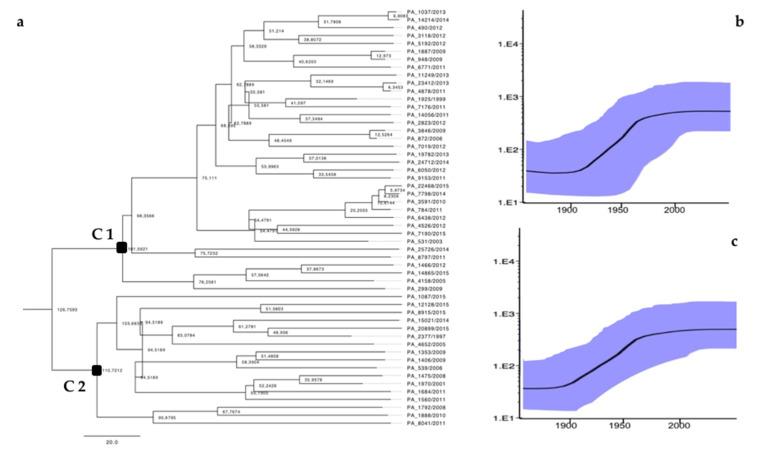
Bayesian maximum clade credibility tree constructed using NS3-NS5B (875 bp) concatenated sequences. The exponential growth demographic model was run for 50 million generations in BEAST 2. (**a**) Bayesian skyline plots of C1 and (**b**) C2 clusters. (**c**) The black line represents the estimated effective number of infections (y-axis) through time (x-axis). The blue shaded area corresponds to the 95% highest posterior density (95% HPD) interval.

The C1 sequences were phylogenetically related to the HCV-4d strains identified in people who belonged to HCV/HIV high-risk groups (IDUs and MSM) living in European countries such as France, Spain, Portugal, and the Netherlands. The C2 strains correlated with the only HCV-4d sequence of a North-African immigrant man available from GenBank. The ancestral relationships were investigated using concatenated NS3-NS5B sequences to increase the number of parsimony-informative sites. The Bayesian MCC tree showed that the common ancestor of analyzed sequences was about 1855 (95% highest posterior density (HPD): 1784–1910). In particular, the C1 sequences group originated in the 1940s (95% HPD: 1922–1955), whereas an older origin of 1915 (95% HPD: 1901–1932) was estimated for C2 sequences. The skyline plots show the exponential increase in HCV-4d infections in the Sicilian population during the 21st century for C1 and C2 strains (Figure 3).

### 3.3. Identification of DAAs Resistance and Immune Escape Mutations

Amino acid analysis of 65 HCV-4d sequences, belonging to C1 and C2 clusters, showed a unique genetic profile that included many RASs as the genetic signature (Table 2).

In the NS5A proteins, we detected mutations as V34I in 66% of strains, F37L in 34%, and W47R in 2%, which were previously described in HCV-4d infected patients at therapeutic failure but not characterized as in vitro, as with the previous isolates [21,27,28]. Multiple RAS combinations were detected in 5% of NS3 genes and 4% of NS5A genes. Two percent of strains showed patterns of both mutations in NS3 and NS5A genes. No drug resistance mutation, identified in NS3 and/or NS5A genes, was associated with C1 or C2 clusters, whereas we found a high probability of generating RAS (m.s. 1) in positions V55A and D168N in NS3 and V34I, F37L, and W47R in NS5A, which were able to confer resistance to VXP and DCV, respectively.

**Figure 4:**
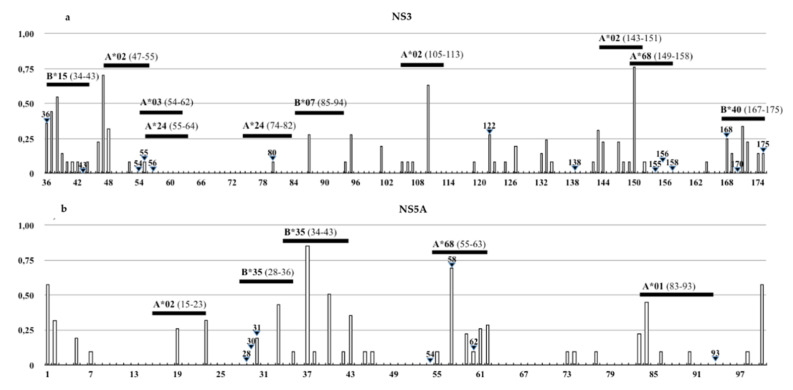
Schematic representation of the amino acid variability in (**a**) NS3 and (**b**) NS5A proteins by the Shannon entropy (ES) value of HCV-4d sequences and the overlapped HLA-I restricted epitopes reported in the IEBD. White vertical bars represent ES values from 0 (low entropy) to 1 (high entropy) and black horizontal bars stand for HLA alleles and HCV epitopes (map positions). Black triangles symbolize residues involved in drug resistance.

Our results showed several amino acid residues, including many RAS positions in NS3 and NS5A proteins, with an ES value >0. As shown in Figure 4, HLA-I-restricted epitopes of NS3 (HLA-A*01:01, A*02:01, A*02:06, A*03:01, A*24:02, A*68:02, B*7:02, B*15:01, B*40:01) and NS5A (HLA-A*02:01, B*35:01) proteins described in the IEBD database were mapped on 4d isolates. The epitope map showed the overlapping of HLA-I-recognized peptides for most of the RASs, excluding 122 and 138 in NS3 and 54 in NS5A. Only the 37th residue of NS5A, inside the epitope map, and the HLA-B*35:01-restricted epitope resulted under positive pressure (dN/dS >1).

To study the impact of amino acid variability on HLA-I recognition, we compared the variation in HLA binding affinity between the wild-type epitope sequences reported in the IEBD and the amino acid sequence of Sicilian HCV-4d strains. The binding prediction analysis, through IC_50_ ANN score variations, revealed that the presence of the RAS V36C, V55A, D168N, and other polymorphic variants (i.e., A87T, L143V, L147M, V150A in NS3 or F36L, F37L/V, R41K, Y43N, P59T, A61S, I63L in NS5A) was able to reduce HLA binding affinity. Mutations that increase IC_50_ ANN scores to >5000 nM were observed in 96% of strains due to the presence of polymorphisms M83T, W84C, and/or S85H/R/N in the NS5A protein, leading to the lack of HLA-A*01:01 recognition. Failure of HLA-I binding was also linked to the presence of Q80K, D168N in NS3 or T56R in NS5A observed in single isolates (Table 3).

## 4. Discussion

HCV genotype 4 dominates in under-developed regions of Africa and the Middle East, where a large population is HIV/HCV co-infected and access to healthcare is limited. Some studies reported the spread of HCV-4d in Southern Europe countries due to the immigration and movement of IDUs [5,7]. According to a phylogenetic analysis conducted in Southern Italy, HCV-4d represents 2.3–7.5% of total HCV infections [9,28]. Phylogenetic reconstructions revealed at least two different HCV genotype 4d transmission patterns involving Sicilian chronically-infected patients: the spread of C1-strains into IDU populations and the simultaneous circulation of different lineages, grouped into the C2-cluster, whose origins remain unknown due to the lack of HCV-4d sequences from other Mediterranean countries in GenBank. The phylodynamic analysis showed the introduction and the exponential growth of HCV-4d cluster 1 in Sicily in the first half of the 20th century, just as reported for HCV-1a and HCV-3a genotypes traditionally associated with drug users [29]. The C2-sequences, instead, showed an older origin in the 1920s, during the Italian colonial period in Eritrea, Somalia, and Libya. At the end of the 19th century, economic difficulties and social crisis caused an increase in migration from Sicily to African colonies and, later, a massive return back to Italy during World War II, which may have contributed to the high prevalence of HCV-4d, and HCV-2c, especially in Southern Italy [8,30].

The study of HCV-4d amino acid variability revealed the presence of RAS and polymorphisms independent from the origin and diffusion routes of the C1 and C2 lineages. HCV-4d lineages in the NS3 protein are characterized by a unique genetic profile that includes the RAS at 36L and 122T, which is involved in reduced susceptibility to SMP and GZP [31,32]. In the NS3 gene, few sequences showed the well-known mutations Q80K and D168E/N associated with resistance to SMP and VXP [33,34]. We found an RAS located near the protease catalytic domain, which did not seem to be directly involved in drug-binding failure but did strengthen the replicative fitness of mutated variants [35]. In the NS5A protein, we identified 30R and 58P as the genetic signature and 34I and 37L were described in HCV-4d-infected patients as a virologic breakthrough [21,27]. Additionally, multiple patterns of RAS in the same protein or both cause a reduction of the SVR rate [36].

The presence of RAS in NS3 and NS5A sequences obtained from DAA naïve patients was described on HCV genotype 1 to 6 [37] and was correlated to the type and number of nucleotide substitutions leading to an amino acid substitution [22]. In particular, a high probability (m.s. 1) of generating a RAS was found in the sequences under study, such as V55A and D168N in NS3 and V34I, F37L, and W47R in NS5A. The viral genome evolves under the pressure of the immune system and DAA therapy due to the overlap between DAA resistance positions and HCV HLA-I restricted epitopes [38]. Epitope binding analysis demonstrated that the Q80K NS3 RAS, spreading in the HCV-1a subtype and sporadically identified in HCV-4d strains, could avoid CD8+ T cell recognition due to the lack of HLA-A*24:02 binding. In the NS5A protein, polymorphisms outside RAS positions, such as the variations at the 83th and 85th residues observed in most isolates, cause the loss of HLA-I A*01:01 binding. HLA-I A*01:01 and A*24:02 alleles are common in European and North African populations, showing a prevalence of 25–30%.

## 5. Conclusions

This is the first study to analyze the circulation of HCV genotype 4 in Sicily, a region in Southern Italy where HCV subtypes co-circulate both in young high-risk subjects and in elderly long-term infected subjects. Intra-subtype variability analysis revealed that HCV-4d strains spreading in Sicily are genetically related to isolates obtained from drugs users in other European countries, carrying mutations able to induce immune escape and resistance to DAAs. This might support the hypothesis of possible variation in genotypes’ geographic distribution and the replacement of endemic genotypes with uncommon genotypes/subtypes, such as HCV-4. In a new approach toward personalized medicine, knowledge of the genetic characterization of HCV isolates prior to therapy could be useful for guiding the selection of the best DAA treatment to increase the probability of recovery. The study of genetic features of common and uncommon HCV genotypes is the first step in the development of new therapies with pan-genotypic activity.

## Figures and Tables

**Table 1:** Demographic and clinical features of HCV-4d infected patients studied. S: Sexual relations, B: Blood, DU: Drug use, U: Unknown.

No. Isolates	Cluster	HIV Co-Infection	Age (Years)			Route of Transmissions			
			<30	30–50	>50	S	B	DU	U
36	C1	11 (30.5%)	4 (11%)	22 (61%)	10 (27%)	3 (0.8%)	2 (0.5%)	14 (39%)	17 (47%)
17	C2	0	0	4 (11.7%)	13 (85%)	0	10 (58%)	0	7 (42%)

**Table 2:** RAS frequencies in NS3 and NS5A proteins, using the resistance prediction rules of the Geno2pheno algorithm. GZP: Grazoprevir, VXP: Voxilaprevir, SMP: Simeprevir, DCV: Daclatasvir, LDV: Ledipasvir.

RAS NS3	Frequency	Resistance to	RAS NS5A	Frequency	Resistance to
36L	92%	GZP, SMP	30R	100%	DCV
122T	92%	SMP	58T	22%	DCV
122A	8%	SMP/PRV	62Q	5%	DCV
36C	6%	GZP	62A	2%	DCV
36A	2%	GZP	31V	2%	DCV, LDV
80K	2%	VXP	58S	2%	DCV
168E	2%	VXP			
168N	2%	VXP/PRV			

**Table 3:** Impact of RASs (bold) and amino acid polymorphisms on HLA-binding affinity evaluated by IC_50_ ANN score. Values <50 nM were considered high affinity, values <500 nM were considered intermediate affinity, values <5000 nM were considered low affinity, and values >5000 nM were considered to have caused a lack of binding.

Epitope Position (Gene/aa.)	HLA-I Allele	IEDB Epitope Sequences	Mutations in Sicilian HCV-4d Strain	Change on HLA-Binding Affinity
NS3/54-62	A*03:01	TVYHGAGTK	V55A	High to intermediate
NS3/55-64	A*24:02	VYHGAGSKTL	V55A	Intermediate to low
NS3/74-82	A*24:02	MYTNVDQDL	Q80K	Lack of binding
NS3/167-175	B*40:02	VDFVPVESM	D168N	Lack of binding
NS5A/34-43	B*35:01	VPFFSCQRGY	F38L	Lack of binding
NS5A/55-63	A*68:02	TTCPCGAQI	T56R	Lack of binding
NS5A/83-93	A*01:01	MWSGTFPINAY	M83T, W84C, S85H/N/R	Lack of binding
